# Hepatic SPARC Expression Is Associated with Inflammasome Activation during the Progression of Non-Alcoholic Fatty Liver Disease in Both Mice and Morbidly Obese Patients

**DOI:** 10.3390/ijms241914843

**Published:** 2023-10-02

**Authors:** Agostina M. Onorato, Lucía Lameroli Mauriz, Juan Bayo, Esteban Fiore, María José Cantero, Barbara Bueloni, Mariana García, Cecilia Lagües, Pedro Martínez-Duartez, Gabriel Menaldi, Nicolas Paleari, Catalina Atorrasagasti, Guillermo D. Mazzolini

**Affiliations:** 1 Gene Therapy Laboratory, Instituto de Investigaciones en Medicina Traslacional, Facultad de Ciencias Biomédicas, CONICET-Universidad Austral, Av. Pte. Perón 1500, Pilar B1629AHJ, Argentina; agos.onorato@live.com.ar (A.M.O.); llameroli-iimt@austral.edu.ar (L.L.M.); jmbayo@hotmail.com (J.B.); estebanjfiore@gmail.com (E.F.); canter.ma@yahoo.com.ar (M.J.C.); barbibueloni14@gmail.com (B.B.); mggarcia24@gmail.com (M.G.); 2 Pathological Anatomy Department, Hospital Universitario Austral, Universidad Austral, Av. Pte. Perón 1500, Pilar B1629AHJ, Argentina; 3 Bariatric and Metabolic Surgery Department, Hospital Universitario Austral, Universidad Austral, Av. Pte. Perón 1500, Pilar B1629AHJ, Argentina; 4 Liver Unit, Hospital Universitario Austral, Universidad Austral, Av. Pte. Perón 1500, Pilar B1629AHJ, Argentina

**Keywords:** non-alcoholic fatty liver disease, secreted protein acidic and rich in cysteine, inflammasome, IL-1β

## Abstract

The severity of non-alcoholic fatty liver disease (NAFLD) ranges from simple steatosis to steatohepatitis, and it is not yet clearly understood which patients will progress to liver fibrosis or cirrhosis. SPARC (Secreted Protein Acidic and Rich in Cysteine) has been involved in NAFLD pathogenesis in mice and humans. The aim of this study was to investigate the role of SPARC in inflammasome activation, and to evaluate the relationship between the hepatic expression of inflammasome genes and the biochemical and histological characteristics of NAFLD in obese patients. In vitro studies were conducted in a macrophage cell line and primary hepatocyte cultures to assess the effect of SPARC on inflammasome. A NAFLD model was established in SPARC knockout (SPARC^−/−^) and SPARC^+/+^ mice to explore inflammasome activation. A hepatic RNAseq database from NAFLD patients was analyzed to identify genes associated with SPARC expression. The results were validated in a prospective cohort of 59 morbidly obese patients with NAFLD undergoing bariatric surgery. Our results reveal that SPARC alone or in combination with saturated fatty acids promoted IL-1β expression in cell cultures. SPARC^−/−^ mice had reduced hepatic inflammasome activation during the progression of NAFLD. NAFLD patients showed increased expression of *SPARC*, *NLRP3*, *CASP1*, and *IL-1*β. Gene ontology analysis revealed that genes positively correlated with SPARC are linked to inflammasome-related pathways during the progression of the disease, enabling the differentiation of patients between steatosis and steatohepatitis. In conclusion, SPARC may play a role in hepatic inflammasome activation in NAFLD.

## 1. Introduction

Non-alcoholic fatty liver disease (NAFLD) is the most common type of chronic liver disease. NAFLD is considered the hepatic manifestation of metabolic syndrome (MS) and is tightly associated with obesity, dyslipidemia, and diabetes. The worldwide prevalence of NAFLD is around 25% in all age groups [1,2]. NAFLD consists of a spectrum of hepatic diseases ranging in severity from fatty acid deposition in hepatocytes (so called steatosis or non-alcoholic fatty liver, NAFL) to a more advanced disease characterized by hepatocyte damage and inflammation (non-alcoholic steatohepatitis, NASH) which can lead to fibrosis, cirrhosis, hepatic failure, or even hepatocellular carcinoma [3]. NAFLD is a histologically defined entity; thus, liver biopsy is the gold standard for diagnosis [4]. Nevertheless, due to patient heterogeneity regarding natural histories and risks of progression, liver biopsy presents limitations and does not always allow an accurate characterization; the correct classification [5], and therefore, a better method for patients’ classification, is needed. The majority of NAFLD patients only present NAFL but 25–40% of patients may develop NASH with or without fibrosis [6]. The time of progression is also variable, and it requires 14 years for each stage of fibrosis and 7 years for each stage of NASH evolution [7,8]. Some patients are considered “rapid progressors”, but identifying and monitoring these individuals is challenging [9]. Thus, understanding the natural history of NAFLD is essential for patient stratification and determining which patients may be at risk of rapid progression.

SPARC (secreted protein acidic and rich in cysteine) is a protein involved in different biological processes including extracellular matrix modulation, tissue renewal, wound healing, response to injury, tissue remodeling, and fibrosis [10]. Several studies demonstrated that SPARC is an important player in the context of obesity, diabetes, and fatty liver disease including advanced hepatic fibrosis [11]. SPARC is also involved in adipogenesis, cellular metabolism, glucose and lipid metabolism, and inflammation [12,13]. We and others described the role of SPARC in liver fibrogenesis and hepatic lipogenesis, demonstrating that the increased hepatic expression of SPARC is associated with liver injury and fibrosis [14,15,16,17,18]. SPARC also has a role in metabolism and homeostasis: SPARC knockout mice (SPARC^−/−^) present increased adipose tissue deposition and impaired glucose homeostasis as the animals age [15,18,19]. In addition, the absence of SPARC worsens high-fat diet-induced diabetes in mice [15]. In a fat diet-induced NASH model, SPARC^−/−^ mice show increased steatosis; nevertheless, reduced hepatic necroinflammation and fibrosis was observed [18]. The role of SPARC in inflammation, particularly in the innate immune response, is not well characterized. It was recently demonstrated that SPARC stimulates a pro-inflammatory macrophage shift in adipose tissue, but its role in the liver has not been explored before [20]. The innate immune system detects endogenous molecules known as damage-associated molecular patterns (DAMPs) released by damaged and dying cells and promotes sterile inflammation through inflammasome activation [21]. Inflammasome signaling plays a key role in NASH pathogenesis and progression. Chronic liver inflammation probably precedes NASH and influences disease progression in certain instances. 

In this study, we wondered whether SPARC might act as a DAMP-promoting inflammasome activation in the in vitro steatosis assay and in high-fat diet-induced NAFLD mice. Additionally, we studied SPARC association with genes of the inflammasome pathway using a public RNAseq database of hepatic tissue from NAFLD patients and searched for new candidate genes expressed along with SPARC in individuals with different stages of NAFLD. Finally, we investigated the hepatic expression of selected genes in a new prospective cohort of morbidly obese patients with NAFLD undergoing bariatric surgery.

## 2. Results

### 2.1. SPARC and Lipotoxic Free Fatty Acids Promote IL-1β Synthesis in Macrophages and Hepatocytes

**Figure 1:**
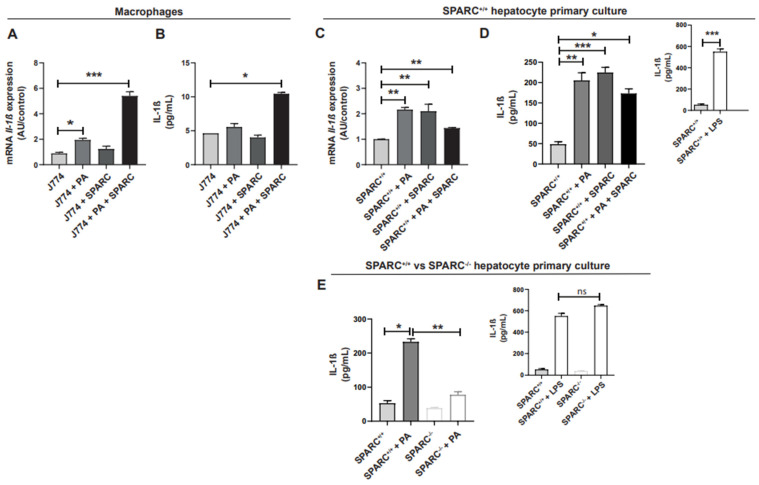
SPARC and palmitic acid (alone or in combination) differently promote *Il-1β* expression and secretion in macrophages and hepatocytes. *Il-1β* expression and secretion in macrophages (J774 cell line) and hepatocyte primary culture exposed to the following: 0.2 mM palmitic acid (PA) for 24 h; 1000 ng/mL LPS for 8 h; and/or 3 µg/mL recombinant SPARC (rSPARC) for 24 h. (**A**) Real-time PCR quantification of *Il-1β* mRNA expression in macrophages (J774 cell line). (**B**) Secreted IL-1β was measured by ELISA in supernatants from the J774 cell culture. (**C**) Real-time PCR quantification of *Il-1β* mRNA expression in *wild type* (SPARC^+/+^) hepatocyte primary cultures exposed to PA, rSPARC, or PA+ rSPARC. (**D**) Secreted IL-1β was measured by ELISA in supernatants from SPARC^+/+^ hepatocyte primary cultures exposed to LPS (upper panel) or to PA, rSPARC, or PA+ rSPARC. (**E**) Secreted IL-1β was measured by ELISA in SPARC^+/+^ and SPARC *knockout* (SPARC^−/−^) hepatocyte primary cultures exposed to PA, rSPARC, or PA+ rSPARC. Upper panel: Quantification of secreted IL-1β in SPARC^+/+^ and SPARC^−/−^ hepatocyte primary cultures exposed to LPS for 8 h. Data are expressed as mean ± SEM, * *p* < 0.05, ** *p* < 0.01, *** *p* < 0.001. Comparison between groups was performed with Kruskal–Wallis and Dunn’s post-test or with Mann–Whitney U test when only two groups were compared. ns, no significant differences. Data are representative of at least three biologically independent replicates.

Inflammasome activation occurs primarily in macrophages when they sense DAMPs [22]. Thus, we analyzed what occurs in vitro with the macrophage cell line J774 when it is co-cultivated with palmitic acid (PA), recombinant SPARC (rSPARC), or a combination of both. PA is a saturated fatty acid with a lipotoxic effect that induces hepatocellular damage during NAFLD [23]. First, PA alone augmented *Il-1β* mRNA expression (Figure 1A); but no difference in IL-1β secretion was observed (Figure 1B). Moreover, rSPARC did not modify mRNA expression nor IL-1β secretion (Figure 1A,B). Nevertheless, the combination of PA and rSPARC greatly increased *Il-1β* mRNA expression (Figure 1A), as well as IL-1β secretion (Figure 1B).

Even though macrophages are the principal cells where inflammasome is activated, it has been demonstrated that hepatocytes are also capable of triggering the inflammasome pathway [24]. Therefore, we next examined whether rSPARC and/or PA could induce *Il-1β* mRNA expression in hepatocyte primary culture from SPARC^+/+^ mice. We observed that *Il-1β* mRNA expression was increased when cells were treated with PA or rSPARC compared with non-treated cells. Interestingly, rSPARC increased *Il-1β* mRNA expression similarly to PA. The combination of PA plus SPARC did not exert synergic effects (Figure 1C). Next, we evaluated IL-1β secretion. The hepatocyte primary culture maintained IL-1β production capacity in response to LPS, a classical inductor of inflammasome (Figure 1D, upper panel). We observed that PA, rSPARC, and the combination of both significantly increased IL-1β secretion (Figure 1D). 

To analyze the effect of the absence of SPARC in IL-1β secretion, we established a hepatocyte primary culture derived from SPARC^−/−^ mice. Both SPARC^+/+^ and SPARC^−/−^ hepatocytes secreted the same basal level of IL-1β and increased secretion in response to LPS in the same way (Figure 1E, upper panel). Interestingly, PA induced IL-1β secretion in SPARC^+/+^ hepatocytes while in SPARC^−/−^ hepatocytes the PA-induced IL-1β secretion was significantly attenuated compared with the PA effect in SPARC^+/+^ hepatocytes (Figure 1E). 

Altogether, these data suggest that both SPARC and/or PA may act as molecules that promote IL-1β expression in macrophages and hepatocytes.

### 2.2. The Absence of SPARC Decreases the Expression of Inflammasome Pathway-Related Genes in the Liver

**Figure 2:**
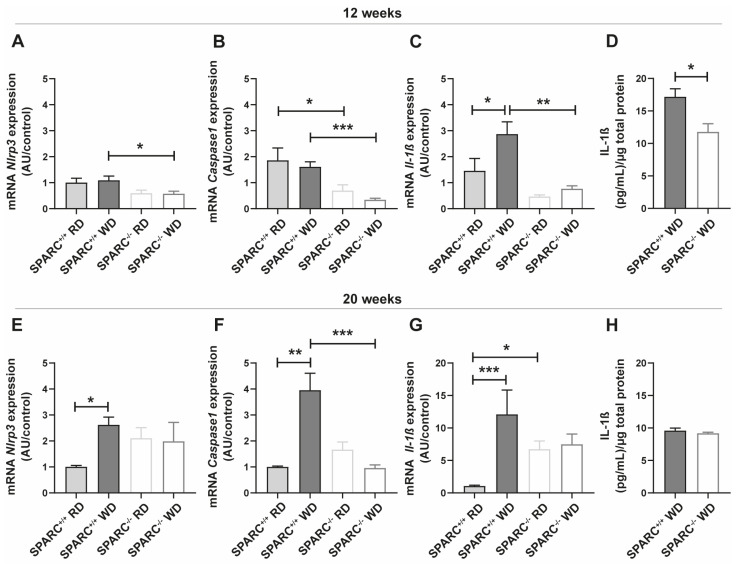
The absence of SPARC decreases the expression of inflammasome pathway-related genes in the liver. Hepatic *Nlrp3*, *Caspase 1* (*Casp1*), and *Il-1β* mRNA expression analysis from SPARC^+/+^ and SPARC^−/−^ mice fed with regular diet (RD) or Western diet (WD) for 12 (**A**–**C**) and 20 (**E**–**G**) weeks. Data are expressed as mean ± SEM, 6 samples per group, * *p* < 0.05, ** *p* < 0.01, *** *p* < 0.001. Comparison between groups was performed with Kruskal–Wallis and Dunn’s post-test. (**D**,**H**) Hepatic expression of IL-1β analyzed at protein level (ELISA) after 12 or 20 weeks of feeding. Comparison between groups was performed with Mann–Whitney U test, * *p* < 0.05.

To confirm the reduced ability to secrete IL-1β demonstrated in SPARC^−/−^ hepatocytes, we analyzed a diet-induced murine model of NAFLD in SPARC^+/+^ and SPARC^−/−^ mice. We previously demonstrated that after 12 weeks of Western diet feeding (WD), SPARC^+/+^ and SPARC^−/−^ mice developed steatosis. After 20 weeks of WD feeding, the animals developed NASH, but a lower inflammation degree was observed in SPARC^−/−^ mice compared to SPARC^+/+^ mice [18]. After 12 weeks, we observed a significantly decreased expression of *Nlrp3* when feeding with WD, the most characterized inflammasome complex in NAFLD [25], and of *Casp1* mRNA expression in regular diet (RD) and WD-fed SPARC^−/−^ mice, compared with SPARC^+/+^ (Figure 2A,B). Western diet induced *Il-1β* expression in SPARC^+/+^ mice, but this effect was not observed in SPARC^−/−^ mice indicating that the absence of SPARC attenuates *Il-1β* expression. Moreover, we observed a significantly decreased *Il-1β* hepatic expression in WD-fed SPARC^−/−^ mice compared with WD-fed SPARC^+/+^ mice (Figure 2C). Remarkably, we observed a significant decrease of hepatic IL-1β in WD-fed SPARC^−/−^ mice by ELISA (Figure 2D). 

However, when the disease progresses to a steatohepatitis stage after 20 weeks of feeding, *Nlrp3* mRNA expression was increased in WD-fed SPARC^+/+^ mice (Figure 2E). In the absence of SPARC, WD did not increase *Nlrp3* expression compared with RD-fed mice. Moreover, a decreased mRNA expression of *Casp1* was observed in WD-fed SPARC^−/−^ mice compared to WD-fed SPARC^+/+^ mice (Figure 2F). WD increased *Il-1β* expression in SPARC^+/+^ mice, but was not able to increase *Il-1β* expression in the absence of SPARC. However, the expression of *Il-1β* was higher in the absence of SPARC, so no significant difference was observed between WD-fed SPARC^+/+^ mice and WD-fed SPARC^−/−^ mice (Figure 2G). When IL-1β was assessed at a protein level, no differences between WD-fed groups were observed (Figure 2H). 

Our results suggest that SPARC may have a role in hepatic innate immune activation. The absence of SPARC attenuates innate immune activation during NAFLD progression, but less during NASH.

### 2.3. SPARC Is Differentially Expressed in NAFLD Patients and SPARC-Positively Correlated Genes Are Associated with Inflammasome-Related Pathways throughout the Progression of NAFLD

We previously observed in a diet-induced NAFLD model that wild type mice increased hepatic SPARC expression during NAFLD development [18]. To assess whether SPARC is differentially expressed in patients with NAFLD, we performed bioinformatic analysis using a public hepatic RNAseq database, GSE130970 [26], which classifies patients at different points of the NAFLD spectrum. Information obtained from the database is summarized in Table S1. Patients were classified according to the stage of disease progression using the NAS histopathological score: healthy (NAS score: 0), fatty liver (NAS score: 1–2), NASH borderline (NAS score: 3–4) and NASH (NAS score: 5–7). Demographic data were obtained from GSE130970 and descriptions of the NAS score and METAVIR score were presented (Supplementary Table S1).

**Figure 3:**
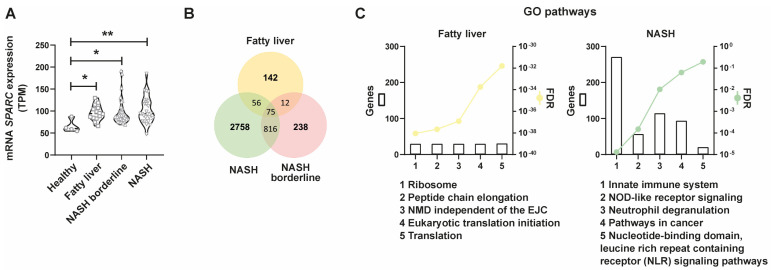
SPARC is differentially expressed in NAFLD patients. SPARC-positively correlated genes are associated with inflammasome-related pathways during NAFLD progression. (**A**) Violin plot representing *SPARC* expression from the RNAseq database GSE130970 between NAFLD groups classified according to the NAFLD activity score (NAS score). Data are expressed in TPM (transcripts per million), * *p* < 0.05, ** *p* < 0.01. Comparison between groups was performed with Kruskal–Wallis and Dunn’s post-test. (**B**) Venn diagrams indicate the number of genes that significantly and positively correlate with the expression of *SPARC* on each patient group of the RNAseq database GSE130970. (**C**) Gene ontology pathway analysis from genes that significantly and positively correlate with *SPARC* on fatty liver and NASH stages. FDR: False Discovery Rate (displayed in logarithmic scale). Genes: number of genes involved on each pathway.

*SPARC* was significantly increased in NAFLD patients compared with healthy patients in the GSE130970 database (Figure 3A). Interestingly, *SPARC* is expressed from early disease stages (Figure 3A). When we compared *SPARC* expression between fatty liver versus healthy patients and NASH versus healthy patients, the strongest differences were observed between NASH and healthy patients. To explore other genes that increased their expressions together with *SPARC*, a global correlation analysis was performed on the RNAseq data, and genes that strongly, positively, and significantly correlate with *SPARC* were determined. The number of genes per group are shown in Venn diagrams (Figure 3B). The gene ontology analysis of biological pathways in the groups of genes previously determined permitted us to recognize differences between the fatty liver and NASH groups of patients. Pathways present in fatty liver patients correspond mainly to protein synthesis or protein expression, whereas NASH patients’ pathways are related to innate immune response, NOD-like receptor signaling, neutrophil degranulation, nucleotide-binding domain, and leucine-rich repeat-containing receptor signaling pathways, among others (Figure 3C). All in all, these data reinforce the fact that SPARC is associated with inflammasome induction.

### 2.4. Inflammasome Gene Expression Analysis in Public Databases of NAFLD Patients

**Figure 4:**
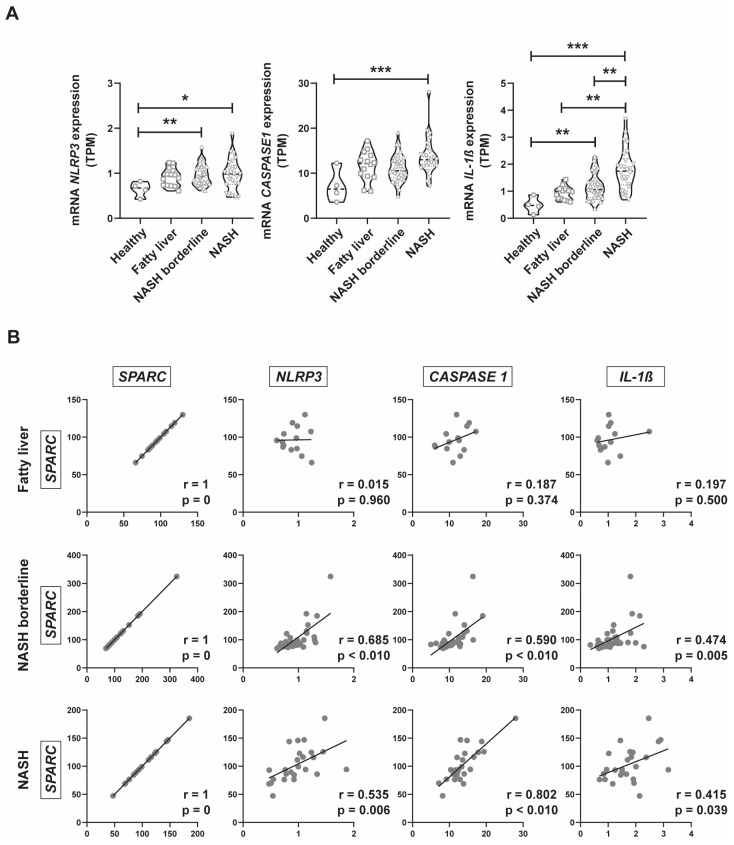
Inflammasome-related gene expression analysis in the public database GSE130970 of NAFLD patients. (**A**) Violin plots representing *NLRP3*, *CASPASE 1* (*CASP1*), and *IL-1β* expression classified according to the NAS histopathological score on the RNAseq database GSE130970. Data are expressed in TPM (transcripts per million), * *p* < 0.05, ** *p* < 0.01, *** *p* < 0.001. Comparison between groups was performed with Kruskal–Wallis and Dunn’s post-test. (**B**) Correlation analysis between *NLRP3*, *CASP1*, and *IL-1β* expression and *SPARC* expression classified according to the NAS histopathological score on the RNAseq database. Pearson test, r: correlation coefficient, ** *p* < 0.01, *** *p* < 0.001.

Considering the results obtained in the gene ontology analysis and in mouse models in vitro and in vivo with inflammasome genes, we decided to evaluate *NLRP3*, *CASP1*, and *IL-1β* expression in the public database. There is a nominally increased expression of each gene while fatty liver disease progresses (Figure 4A). In particular, IL-1β is expressed differently in each stage of the disease, increasing its expression as the disease progresses from NAFL to NASH (Figure 4A). Then, we performed a correlation analysis to assess whether there is an association between SPARC and inflammasome genes. As a result, we observed that in NASH borderline and NASH stages there is a positive and statistically significant correlation between SPARC and *NLRP3*, *CASP1*, and *IL-1β* genes (Figure 4B). These results highlight that both inflammasome gene expression and SPARC expression are correlated during NAFLD progression.

### 2.5. AKR1B10 and FABP5 Positively Correlate with SPARC Expression

To identify other candidate genes which positively correlated with *SPARC* in NASH patients and whose expression could be different between patients with fatty liver and NASH, we studied the 2758 genes that present a strong correlation with SPARC (Figure 3B) and explored which ones were differentially expressed in NASH and NAFL stages. We reached out to 74 candidates, which would allow us to differentiate patients between these two stages. From those 74 genes, we selected two final candidates overexpressed in NASH but not in fatty liver: *AKR1B10* (Aldo-Keto Reductase Family 1 Member B10) and *FABP5* (Fatty Acid Binding Protein 5). Both genes encode for secreted proteins (validated by the SecretomeP-2.0 program that predicts secretion) [27] that could be detectable in serum, an advantage over invasive biomarkers. On the other hand, previous works demonstrated their importance in the physiopathology of NAFLD [28,29,30,31]. 

**Figure 5:**
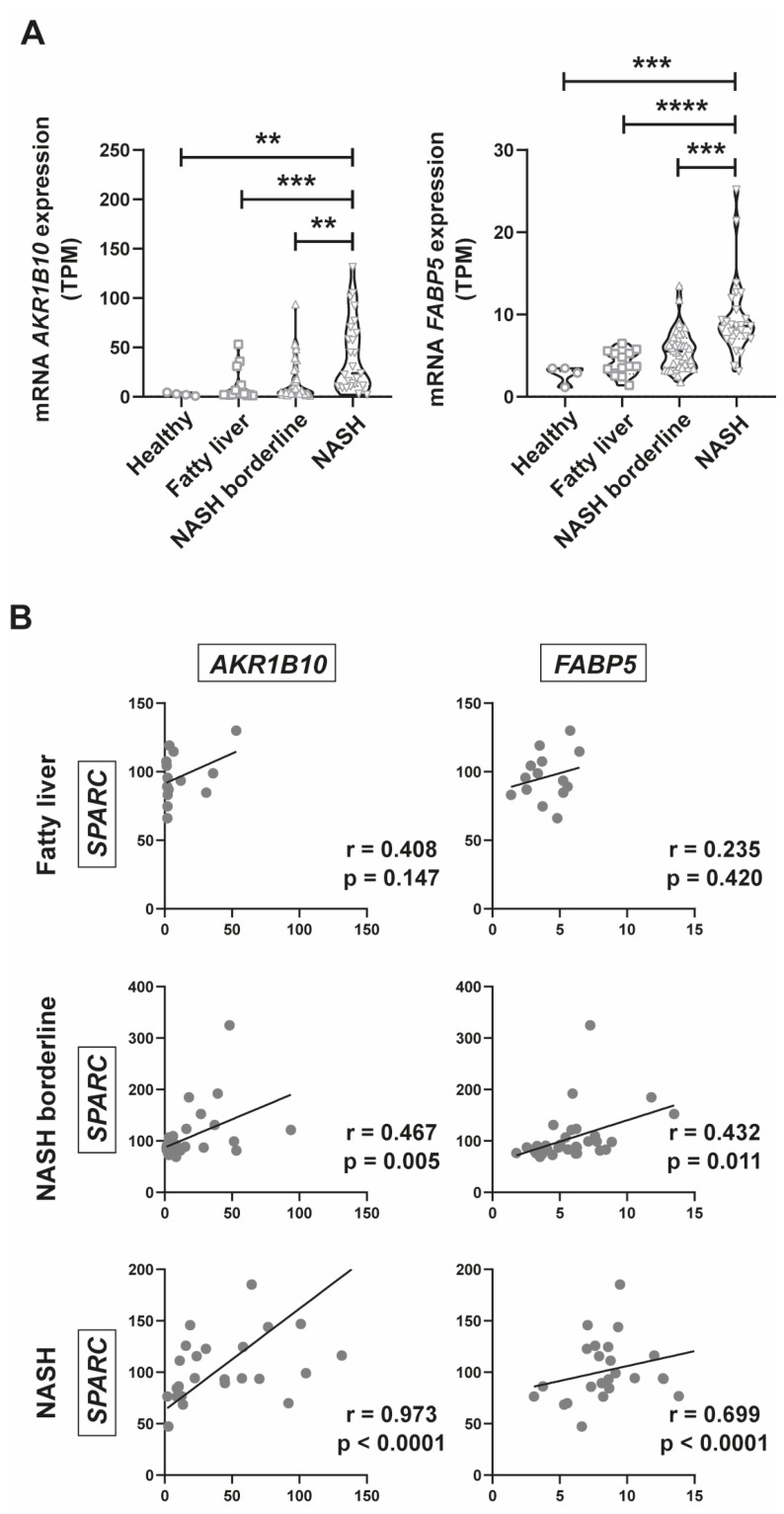
*AKR1B10* and *FABP5* expression analysis in public databases GSE130970. (**A**) Violin plots representing *AKR1B10* and *FABP5* mRNA expression classified according to the NAS histopathological score on the RNAseq database GSE130970. Data are expressed in TPM (transcripts per million), ** *p* < 0.01, *** *p* < 0.001, **** *p* < 0.0001. Comparison between groups was performed with Kruskal–Wallis and Dunn’s post-test. (**B**) Correlation analysis between *AKR1B10* and *FABP5* expression and SPARC expression classified according to the NAS histopathological score on the RNAseq database GSE130970. Pearson test, r: correlation coefficient.

First, using the public database, we assessed gene expression of *AKR1B10* and *FABP5* in groups of patients divided by NAS score. We observed that along with the progression of fatty liver disease, their gene expression is significantly increased (Figure 5A). In addition, we performed a correlation analysis between SPARC and *AKR1B10* and *FABP5* expression in fatty liver, NASH borderline, and NASH groups. Remarkably, we observed a positive and significant correlation between *SPARC* and these genes in NASH borderline and NASH groups (Figure 5B). Altogether, our data suggest that *AKR1B10* and *FABP5* may be good candidate target genes to differentiate between NAFLD stages.

### 2.6. SPARC expression positively correlates with CASP1, IL-1β, AKR1B10, and FABP5 Expression in a New Prospective Patient Cohort

To confirm previous results in a prospective new patients’ cohort, we obtained liver biopsies paired with serum samples from obese patients undergoing bariatric surgery at our institution (HUA cohort). This cohort is composed by 59 patients including all NAFL disease stages, as well as “healthy” patients, according to NAS histopathological score criteria. This patient population is characterized by the presence of mild fibrosis. Demographic, histopathological, and biochemical data are presented in Table 1. 

**Figure 6:**
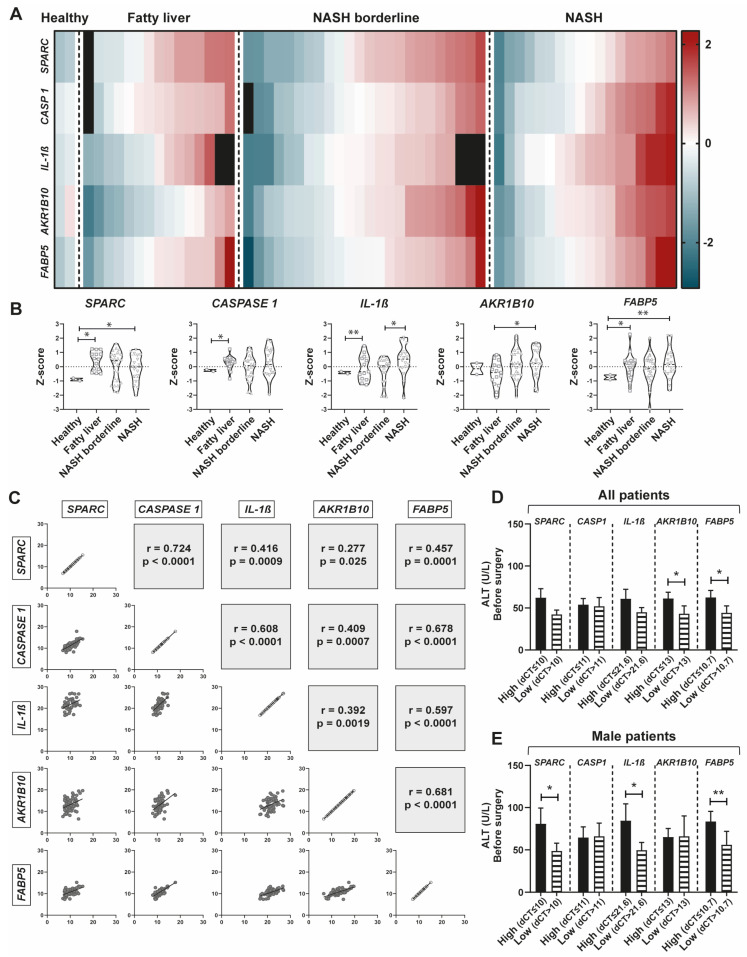
*SPARC*, *CASPASE 1*, *IL-1β*, *AKR1B10*, and *FABP5* hepatic mRNA expression and correlation analysis in the HUA patient cohort with different stages of NAFLD. (**A**) qPCR quantification of hepatic *SPARC, CASPASE 1 (CASP1), IL-1β, AKRB10*, and *FABP5* mRNA expression in hepatic tissue from patients at the time of the surgery. Heatmap visualization of z-score calculated from qPCR analysis. Negative values (blue) represent low expression, positive values (red) represent high expression. Zero (white) represents no expression variability. Black spaces represent excluded values, including outliers and no gene expression. (**B**) Violin plots representing *SPARC*, *CASP1*, *IL-1β, AKR1B10*, and *FABP5* expression classified according to the NAS histopathological score. Data are expressed in z-score. (**C**) Correlation matrix between *SPARC, CASP1, IL-1β, AKR1B10*, and *FABP5* expression from all patients. Spearman test. (**D**,**E**) ALT (U/L) values from all patients or men’s sub-cohort between 1 to 6 months prior to surgery. Patients were divided in two groups: those who have high *SPARC, CASP1, IL-1β, AKR1B10*, or *FABP5* expression and those who have low expression. Division was performed considering the mean of each gene’s expression (dCT ≤ 10 and dCT > 10, for *SPARC*; dCT ≤ 11 and dCT > 11, for *CASP1*; dCT ≤ 21.6 and dCT > 21.6, for *IL-1β*; dCT ≤ 13 and dCT > 13, for *AKR1B10*; dCT ≤ 10.7 and dCT > 10.7, for *FABP5*). Data are expressed as mean ± SEM. * *p* < 0.05, ** *p* < 0.01, Mann–Whitney U test.

We studied *SPARC*, *CASP1*, *IL-1β*, *AKR1B10*, and *FABP5* mRNA liver expression, by qPCR, in healthy and NAFLD patient groups. Interestingly, for each gene we observed that there were patients with low expression or high expression, independently of NAFLD group (Figure 6A,B). Z-score graphs show that SPARC expression was increased in NAFLD patient groups compared to healthy individuals. Moreover, the expression of *CASP1* and *IL-1β* was increased in NAFL patients compared to healthy patients, while in patients with advanced NAFL disease there was an enrichment of subjects presenting higher expression. Further, we assessed *AKR1B10* and *FABP5* mRNA expression in the HUA cohort. Interestingly, increased expression of *AKR1B10* was observed in NASH patients compared with NAFL, while *FABP5* expression was consistently increased as the disease progresses compared with healthy obese patients (Figure 6A,B). To evaluate the relationship between all analyzed genes, we performed a correlation analysis between *SPARC*, *CASP1, IL-1β*, *AKR1B10*, and *FABP5* expression. Noticeably, these genes positively and significantly correlate to each other as shown in the correlation matrix (Figure 6C), suggesting that there is an association between them and confirming what we previously observed in public NAFLD databases.

Serum alanine aminotransferase (ALT) levels were measured in each patient between 6 to 1 months before surgery. We wondered whether patients with higher levels of ALT were the ones that presented increased expression of *SPARC*, *CASP1*, *IL-1β*, *AKR1B10*, and *FABP5*. Therefore, we grouped patients depending on whether they had high or low expression of each gene (considering the mean expression value). No demographic and histopathological differences were observed between low and high subgroup patients (Supplementary Table S2). Patients in the high expression group showed a nominal increase in ALT values compared to the low expression group for each gene. For *AKR1B10* and *FABP5* mRNA expression, significant differences were observed between the high and low expression patients’ groups (Figure 6D). Interestingly, when we considered only the sub-cohort of men (50.8% of the population), patients in the *SPARC* and *IL-1β* low expression group also had a significant decrease in ALT values compared to the high expression group (Figure 6E). 

All in all, the data obtained from our prospective patient cohort suggest that independently of the histopathological classification of patients, the increased expression of these genes was associated with higher hepatocellular damage.

## 3. Discussion

SPARC is implicated in numerous biological processes and has been shown to play a role in NAFLD, encompassing wound-healing responses to injury, tissue remodeling, and fibrosis, as well as glucose homeostasis and lipid metabolism [11,13,32]. SPARC is expressed and secreted by hepatic stellate cells (HSC) and hepatic endothelial cells (LSECs) during tissue damage [33,34,35,36]. This study offers pivotal insights by demonstrating that SPARC also contributes to inflammasome activation in a paracrine manner. It was previously demonstrated that Follistatin-like 1 (FSTL-1), a protein belonging to the SPARC protein family, acts on the NLRP3 inflammasome to promote IL-1β secretion in macrophages [37]. However, the role for SPARC on inflammasome activation was not reported until now. In adipose tissue, it was recently observed that SPARC converted anti-inflammatory macrophages into a pro-inflammatory phenotype by inducing interferon β (INF-β) in a TLR4-dependent manner [20]. In this work, the role of SPARC on inflammasome activation in macrophages and hepatocytes was explored for the first time. In vitro studies performed in a macrophage cell line indicate that the combination of recombinant SPARC plus palmitic acid (PA) increases IL-1β expression and secretion indicating the activation of inflammasome. Cai et al. previously demonstrated that PA could act as a kind of damage-associated molecular pattern to elevate the messenger RNA and protein expression levels of NLRP3, ASC, and caspase-1 stimulating IL-1β secretion in Kupffer cells [38]. Here we observed that PA or SPARC alone are not able to significantly increase IL-1β secretion, but in combination, PA plus SPARC induces IL-1β secretion. Inflammasome activation may need more than one signal. Hepatocytes may also activate inflammasome pathways in response to PA. Czak et al. demonstrated that a PA-treated hepatocyte cell line activates the inflammasome and induces sensitization to the LPS-induced IL-1β release in hepatocytes [39]. Interestingly, we demonstrated that recombinant SPARC increased IL-1β expression and secretion in hepatocytes in the same way that PA does. Single rSPARC treatment has no effect in macrophage cell lines but it is sufficient to stimulate IL-1β expression and secretion in hepatocyte primary culture. One possible explanation for this observation is that the primary culture associated stress may induce the release of other DAMPs that may contribute to activation. In fact, rSPARC alone is not enough to activate IL-1β secretion in the J774 macrophage cell line. All in all, our results suggested that SPARC may act as a new DAMP that contributes to the activation of the innate immune response in both macrophages and hepatocytes. Different matricellular proteins have been implicated in inflammatory and restorative responses following tissue damage, and many of them act as danger signals [40,41]. SPARC is a non-structural matricellular protein, which is upregulated upon liver injury. Previous studies from our group demonstrated in two NAFLD-murine models that hepatic *Sparc* expression increases during disease progression [17,18]. Moreover, SPARC acts as an acute phase reactant in both acute and chronic hepatic disorders [15]. Growing evidence suggests that inflammasome activation, particularly NLRP3, plays a significant role in several acute and chronic liver diseases, including NAFLD [42]. This promoted us to investigate the association between SPARC and inflammasome activation in the context of NAFLD. Increased SPARC secretion, mainly by HSCs and LSECs, in response to tissue injury towards the extracellular environment may modulate inflammasome activation in hepatic macrophages but also in hepatocytes. We previously described that SPARC^−/−^ mice had less inflammation despite higher steatosis compared to SPARC^+/+^ in a diet-induced NAFLD mice model [18]. SPARC acts as an adipogenesis inhibitor in adipose tissue, and its absence increases adipose deposits with the concomitant increase in FFA in the context of liver steatosis [43]. Despite its association with increased hepatic steatosis, SPARC^−/−^ mice show less hepatic inflammation in later stages of the disease [18]. In this work, we found that after 12 weeks of feeding, SPARC^−/−^ mice had decreased inflammasome activation, as evidenced by a decreased expression of *Nlrp3*, *Casp1*, and synthesis of IL-1β in hepatic tissue, suggesting that the absence of SPARC attenuates the inflammasome activation in hepatic tissue. Inflammasome activation in chronic diseases such as NAFLD induces a deleterious effect on the liver and on steatohepatitis development. Indeed, *Nlrp3* knockout mice present less diet-induced hepatic inflammation and fibrosis [25]. The inhibition of inflammasome activation in the absence of SPARC during the initial stages of the disease may explain, at least partially, the attenuation of steatohepatitis severity. However, after 20 weeks of feeding and as the disease progresses, reduction in hepatic IL-1β levels was not observed compared with SPARC^+/+^ mice. Considering that tissue damage produces several molecules that may trigger inflammasome activation, it is reasonable to think that in advanced disease stages inflammasome activation depends on multiple danger-associated molecules and occurs despite the absence of SPARC. 

Next, we wondered whether varying levels of hepatic SPARC expression could serve to distinguish patients across different stages of NAFLD. To this end, we explored a public database of patients with NAFLD, and found that *SPARC* expression is increased in the livers of patients with different stages of the disease compared to patients without histological evidence of NAFLD. *SPARC* expression was increased in human cirrhotic livers [14,44]. Moreover, in a previous study of our group, *SPARC* expression was studied in hepatic biopsies from morbidly obese patients. Patients with higher SPARC expression levels presented more hepatocellular damage and increased mRNA expression of pro-fibrogenic factors [18]. In a public database, we observed that *SPARC* expression is increased in NAFLD patients compared with obese patients without histological evidence of NAFLD, although no significant differences were observed between individuals with NAFL compared with those with NASH. These results were confirmed in a prospective cohort of obese patients undergoing bariatric surgery at our institution (HUA cohort). In line with our results, a recent study applying single-cell transcriptomics identified that *SMOC2*, a member of the SPARC family, showed increased expression in hepatic stellate cells during NAFLD development and in plasma, suggesting that SMOC has potential as a new diagnostic biomarker for NASH patients [45]. 

As previously mentioned, the activation of inflammasome plays a crucial role in the progression of NAFLD, as its activation initiates tissue damage, inflammation, and the development of liver fibrosis [24,46,47]. In the present study, hepatic *NLRP3*, *CASP1*, and *IL-1β* expression was increased in patients with biopsy-proven NASH. Particularly, *CASP1* and *IL-1β* expression was significantly different between NAFL and NASH patients. In line with our previous observation, hepatic SPARC expression correlated with the expression of these inflammasome-related genes. When analyzing public data, the strength of the correlation depends on the stage of NAFLD progression; in the GSE130970 database a significantly positive correlation was observed in NASH and NASH borderline stages. Moreover, we found a set of candidate genes that positively correlated with *SPARC* and simultaneously presented differential expression between fatty liver and NASH patients. Among them, *AKR1B10* and *FABP5* were of particular interest. Both genes encode for proteins involved in NAFLD-related pathways and were detected not only in hepatic tissue but also in serum. *AKR1B10* has been previously identified in an in silico secretome analysis and proposed as a biomarker for NAFLD [29]. *FABP5* has been also proposed as a marker of NAFL to NASH progression [48]. We observed both in public databases and in our patient cohort that their expression was increased in patients with NASH. Moreover, over-expression of *SPARC* strongly correlated with the expression of these genes and confirms its implication in NAFLD progression.

Although histological evaluation remains the gold standard for diagnosing progressive NASH, it has several disadvantages, such as sampling error and operator variability [49,50]. Therefore, it is necessary to establish other parameters to characterize the degree of damage in liver biopsies. Even more, grouping patients based on histology can lead to improper clinical management of patients [51]. Here we show that in each group of patients (NAFL, NASH borderline, and NASH) different patterns of gene expression were detected, as we observed that some patients had increased *SPARC*, *CASP1*, *IL-1β*, *AKR1B10*, and *FABP5,* while others showed decreased expression. Therefore, data obtained from our prospective patient cohort suggest that independently of the histopathological classification of patients, the increased expression of these genes was associated with higher hepatocellular damage. 

The single evaluation of hepatic transaminases is unsuitable for non-invasive screening or diagnosis of NAFL or NASH in morbidly obese patients [52]. Increased ALT is closely related to fat accumulation and hepatocellular damage and, consequently, it has been used as a marker of NAFLD progression [53]. In this line, we found in the HUA patient cohort that patients expressing low *SPARC*, *IL-1β*, *AKR1B10*, and *FABP5* presented reduced ALT levels compared with the high expression groups. Our results indicate an association between the expression of *SPARC, IL-1β*, *AKR1B10*, and *FABP5,* and increased ALT levels before surgery. The analysis of these genes in liver biopsies could help to better understand and characterize patients with NAFLD. 

Overall, this study sheds light on the role of SPARC in NAFLD pathogenesis and its association with pathway genes. In addition, we demonstrated a link between SPARC and AKR1B10 and FABP5 genes, which could aid in better stratifying NAFLD patients and identifying those at risk of disease progression. Further research is warranted to unravel the underlying mechanisms and explore the potential therapeutic applications of targeting SPARC and its associated genes in NAFLD. In the future, considering genetic data may complement the histopathological grade analysis to improve patient stratification. 

## 4. Materials and Methods

### 4.1. J774 Culture, Primary Hepatocyte Isolation, and In Vitro Steatosis Assay

For in vitro experiments, the J774 macrophage cell line (*ATCC*) and primary hepatocyte cultures were cultured at 37 °C and 5% CO_2_. J774 macrophages were cultured in Roswell Park Memorial Institute medium (RPMI) (Life Technologies, Carlsbad, CA, USA) 10% fetal bovine serum (FBS) (Internegocios S.A., Mercedes, Argentina, with 2 mM glutamine, 100 µg/mL streptomycin, and 100 µg/mL penicillin. Hepatocytes were isolated by collagenase perfusion [54] from 8–10 weeks-old SPARC^+/+^ and SPARC^−/−^ mice. Hepatocytes were grown in phenol red-free Dulbecco’s modified Eagle’s medium (DMEM) 10% FBS, with 2 mM glutamine, 100 µg/mL streptomycin, and 100 µg/mL penicillin. After 3 h, the medium was replaced with DMEM/F12 (70% DMEM + 30% F12) 10% SFB. Two days after seeding, cells were incubated overnight (ON) with 0.2 mM of palmitic acid (PA) (Invitrogen) diluted in DMEM 2% FBS which induced lipotoxicity without generating cell death [55]. Alternatively, cells were cultured ON with 3 µg/mL recombinant SPARC (rSPARC) (SinoBiological, Wayne, PA, USA) or a combination of rSPARC and PA. Also, to measure the IL-1β protein level on primary hepatocyte cultures, cells were cultured with 1000 ng/mL LPS for 8 h.

### 4.2. Animal Experimental Design

In vivo experiments were performed in male C57BL/6J mice and SPARC^−/−^ mice on a C57BL/6 background (Jackson Laboratory, Bar Harbor, ME, USA). Animals were maintained in a temperature-controlled environment on a 12 h/12 h light/dark cycle. Food and water were available ad libitum. Six-weeks-aged SPARC^−/−^ and SPARC^+/+^ mice were randomized in four treatment groups (6–8 mice per group): group 1) SPARC^+/+^ fed with high fat diet and 50 g/L sucrose was added to drinking water (termed Western diet, WD); group 2) SPARC^+/+^ fed with regular laboratory chow diet (RD); group 3) SPARC^−/−^ fed with WD; group 4) SPARC^−/−^ fed with RD [56,57]. The WD contains calcium caseinate (200 g/kg), vitamin mixture (10 g/kg), cellulose (50 g/kg), animal fat (250 g/kg), vitamin A (1 mL/kg), choline bitartrate (2.5 g/kg), and maltodextrin (451.5 g/kg). The mixture of vitamins and minerals was prepared according to the recommendations of the AIN 9325. Animals in each group were euthanized after 12 or 20 weeks of feeding. All protocols dealing with animals were reviewed and approved by the Austral University Animal Studies Committee. This study followed the guidelines outlined in the National Institutes of Health Guide for the Care and Use of Laboratory Animals.

### 4.3. Quantitative Real-Time PCR (qPCR)

Cell cultures and murine liver or human liver tissues were homogenized, and total RNA was extracted using Trizol reagent (Sigma-Aldrich, St. Louis, MO, USA). RNA (1–2 µg) was reverse-transcribed with 1 µL RevertAid (200 U/µL) (Life Technologies, Carlsbad, CA, USA ) using 2 µL of Oligo dT primers (250 ng/µL) (*PB-L*). cDNAs were subjected to qPCR. To measure *Sparc*, *Nlrp3*, *Caspase 1* (*Casp1*), and Il-1β, in both cell cultures and murine liver tissues, cDNA samples were diluted 0.5:5 in nuclease free H_2_O. To measure *SPARC*, *NLRP3*, *CASPASE 1* (*CASP1*), IL-1β, *AKR1B10*, and *FABP5* in human liver tissues, cDNA samples were diluted 0.3:5 in nuclease free H_2_O. For every 5 µL of sample dilution, 10 µL of Master Mix qPCR 2.0 (Sybr/ROX) (PB-L, Buenos Aires, Argentina) was used. All PCR amplifications were carried out using 40 cycles of 95 °C for 30 s, 55–56 °C for 30 s, and 72 °C for 30 s. At the end of the PCR reaction a melting curve was performed. mRNA levels of *Gapdh* or 18S were used as endogenous control. All data were analyzed using AriaMx Real-Time PCR System Software (Agilent, Palo alto, CA, USA). Changes in gene expression were expressed as 2^ΔΔCt^ referred to as fold change compared to the mean expression quantified in controls or only as Δt (delta Ct) for human qPCRs. See supplementary Table S3 for the list of primers utilized for qPCR.

### 4.4. ELISA

IL-1β concentration was determined by *DuoSet ELISA* (R&D Systems, Minneapolis, MN, USA). Plates pre-coated with monoclonal antibodies were blocked by adding Reagent Diluent, washed with wash buffer, and incubated with samples. To determine the protein concentration, BCA (Pierce Biotechnology, Rockford, IL, USA) was used.

### 4.5. Bioinformatic Analysis

The GSE130970 [26] database was selected from the Gene Expression Omnibus (GEO); it contains transcriptomic data of liver biopsies from obese patients that cover the NAFLD spectrum obtained by RNAseq assays. The GSE130970 database contains the expression profile of 78 liver biopsies (4 histologically normal and 74 from NAFLD patients). Patients were classified according to their NAS score [58] in four groups: healthy (individuals with no evidence of liver damage), fatty liver (NAS 1–2), NASH borderline (NAS 3–4), and NASH (NAS ≥ 5).

Gene expressions of *SPARC*, *CASP1*, *IL-1β, AKR1B10*, and *FABP5* were evaluated in each group. Correlation analysis between *SPARC* and all the genes in the databases were performed with *RStudio.*

Ontological analyses were performed with Toppfun (Toppgene). Pathways with significant FDRs (False Discovery Rates) were selected. 

### 4.6. Patients’ Cohort and Study Protocol

A cross-sectional prospective observational study was performed. The study population consisted of 59 Caucasian obese patients undergoing bariatric surgery (BAS) at Hospital Universitario Austral, Argentina. Liver biopsies were taken by decision of the medical team from bariatric surgery service in patients with evidence of liver damage at the procedure time, as for example: changes in the coloration and texture of the liver, severe steatosis, hepatomegaly, signs of chronic liver injury of fibrosis or cirrhosis, signs of portal hypertension. Men or women >18 and ≤65 years with a body mass index (BMI) ≥35 kg/m^2^, with obesity maintained for more than 5 years in which other treatments failed, were included in this study. Patients with a history of alcohol consumption ≥20 g/day in women and ≥30 g/day in men, patients with positive serology for hepatitis B virus and hepatitis C virus, and also patients who consumed hepatotoxic drugs, with autoimmune or genetic liver disease, were not included in this study. All patients were informed about the additional risks of a wedge liver biopsy during the bariatric procedure. Liver specimens were split and stored in either 10% (*v*/*v*) formalin solution for subsequent histological examination or in Trizol reagent (Sigma-Aldrich, St. Louis, MO, USA) for RNA isolation. Serum from each patient was obtained at the time of surgery. All patients provided written informed consent before participating in this study, approved by protocol No.16-049 Local Ethical Committee (Austral University).

### 4.7. Clinical–Biochemical Assessment and Histopathology

Anthropometric parameters, such as age, sex (M/F), and BMI (kg/m^2^) were scored during the baseline visit. Blood samples were collected between 1–6 months prior to surgery and at the time of the surgery for assessment of liver biochemistry and glucose. Surrogate markers’ scores of liver fibrosis were calculated as described by Sumida for FIB-4 [59]. Liver wedge biopsies were performed, and liver tissue was fixed in 10% formalin and embedded in paraffin. Hematoxylin and eosin liver specimens were evaluated by light microscopy. An experienced pathologist assessed steatosis, inflammation, and ballooning in a blind fashion. Steatosis, inflammation, and ballooning were scored based on the NAS score. For liver fibrosis assessment, sections were stained with Masson’s trichrome, and fibrosis diagnosis was determined in accordance with Brunt fibrosis criteria [58].

### 4.8. Statistical Analysis

All data were analyzed using Prism version 8.0.1 (*GraphPad*). Data are reported as arithmetic means ± SEM. Depending on the data distribution and number of groups, a statistical analysis with the appropriate test was determined. Two-way ANOVA with Bonferroni correction for multiple comparison, one-way ANOVA Kruskal–Wallis test with Dunn’s multiple comparisons post-test, or the Mann–Whitney U test were applied. For all analyses, a *p*-value < 0.05 was considered statistically significant (*p* < 0.05 (*), *p* < 0.01 (**), *p* < 0.001 (***)). For correlation analysis, Spearman rank correlation or Pearson correlation was applied. 

## Figures and Tables

**Table 1:** Demographic, histopathological, and clinical features of a single-center prospective cohort (HUA cohort).

**Hospital Universitario Austral cohort**	**Variable**		**Healthy**	**Fatty liver**	**NASH borderline**	**NASH**
	*n* (Total = 59)		2 (3.4%)	15 (25.4%)	24 (40.7%)	18 (30.5%)
	Sex	M	1 (50%)	6 (40%)	13 (54.2%)	10 (55.6%)
		F	1 (50%)	9 (60%)	11 (45.8%)	8 (44.4%)
	Age (mean) (SD)	M	60 (0.0)	47.7 (12.5)	47.2 (9.6)	42.1 (4.5)
		F	43 (0.0)	46.7 (10.4)	40.3 (12.3)	47.8 (7.8)
	Height (mean in metres) (SD)	M	1.65 (0.0)	1.75 (0.05)	1.74 (0.06)	1.75 (0.07)
		F	1.54 (0.0)	1.59 (0.03)	1.60 (0.07)	1.61 (0.03)
	Weight (mean in Kg) (SD)	M	117 (0.0)	121.5 (25.5)	127.2 (22.0)	123.2 (20.1)
		F	94 (0.0)	100.2 (16.1)	108.1 (17.5)	105.4 (9.1)
	Body mass index (BMI)	M	42.8 (0.0)	40.0 (8.7)	42.4 (9.8)	40.4 (8.1)
		F	39.6 (0.0)	39.3 (5.7)	42.5 (6.7)	40.8 (2.7)
	**NAFLD activity score**		**0**	**1 & 2**	**3 & 4**	**5, 6 & 7**
	Steatosis grade	0	2 (100%)	3 (20%)	0	0
		1	0	12 (80%)	16 (66.7%)	2 (11.1%)
		2	0	0	8 (33.3%)	8 (44.4%)
		3	0	0	0	8 (44.4%)
	Lobular inflammation grade	0	2 (100%)	7 (46.7%)	1 (4.2%)	0
		1	0	7 (46.7%)	22 (91.6%)	5 (27.8%)
		2	0	1 (6.6%)	1 (4.2%)	12 (66.7%)
		3	0	0	0	1 (5.5%)
	Cytological ballooning grade	0	2 (100%)	11 (73.3%)	0	0
		1	0	4 (26.7%)	21 (87.5%)	6 (33.3%)
		2	0	0	3 (12.5%)	12 (66.7%)
	**Fibrosis stage**					
	0		2 (100%)	8 (53.3%)	5 (20.8%)	5 (27.8%)
	1		0	3 (20%)	11 (45.8%)	9 (50%)
	2		0	0	2 (8.3%)	1 (5.5%)
	3		0	0	3 (12.5%)	3 (16.7%)
	4		0	0	1 (4.3%)	0
	No fibrosis data available		0	4 (26.7%)	2 (8.3%)	0
	**Biochemical analysis (Serum sample taken at the time of bariatric surgery)**					
	Albumin (mean in g/dL) (SD)	M	3.74 (0)	4.14 (0.3)	3.89 (0.7)	4.27 (0.4)
		F	4.69 (0)	3.97 (0.5)	4.08 (0.4)	3.40 (0.8)
	Alkaline phosphatase (mean in U/L) (SD)	M	42.3 (0)	63.86 (8.7)	57.00 (23.6)	41.92 (22.9)
		F	99.1 (0)	53.26 (21.3)	59.42 (19.4)	56.48 (31.2)
	ALT (mean in U/L) (SD)	M	3 (0)	13.88 (7.6)	18.12 (14.9)	20.16 (12.7)
		F	9.6 (0)	21.10 (32.2)	11.01 (2.7)	13.95 (9.7)
	AST (mean in U/L) (SD)	M	12.9 (0)	30.40 (10.3)	32.68 (14.6)	54.36 (30.2)
		F	16 (0)	44.93 (47.2)	49.64 (46.9)	47.83 (45.3)
	Direct bilirubin (mean in mg/dL) (SD)	M	0.24 (0)	0.28 (0.09)	0.29 (0.14)	0.24 (0.07)
		F	0.38 (0)	0.25 (0.13)	0.21 (0.1)	0.22 (0.1)
	Total bilirubin (mean in mg/dL) (SD)	M	0.57 (0)	0.75 (0.2)	0.74 (0.43)	0.71 (0.28)
		F	1.03 (0)	0.59 (0.25)	0.46 (0.24)	0.47 (0.26)
	Cholesterol (mean in mg/dL) (SD)	M	137.4 (0)	143.6 (41.3)	134.4 (37.4)	144.5 (27.0)
		F	206 (0)	161.0 (35.5)	147.7 (20.1)	144.2 (49.2)
	Blood glucose (mean in mg/dL) (SD)	M	95.1 (0)	119 (36.4)	107.0 (31.1)	112.3 (21.7)
		F	104.2 (0)	102.3 (23.2)	126.3 (33.5)	128.1 (41.7)
	Prothrombin time (mean in g/dL) (SD)	M	6.36 (0)	7.47 (0.4)	6.85 (1.4)	8.20 (1.7)
		F	8.8 (0)	7.47 (0.9)	7.68 (1.7)	6.69 (1.7)
	No biochemical data available (*n*)		0	3	2	0

Data from morbidly obese patients undergoing bariatric surgery at the Hospital Universitario Austral (HUA). Liver biopsy and serum samples were collected at the time of surgery. Patients were classified in four groups according to the NAS histopathological score: healthy (NAS score 0), fatty liver (NAS score 1–2), NASH borderline (NAS score 3–4), and NASH (NAS score 5–7). Table provides demographic information: sex (M: masculine or F: feminine), age (mean ± SD), and Body Mass Index (BMI). Clinicopathological characteristics were provided for each patient group. ALT: alanine transaminase; AST: aspartate transaminase.

## Data Availability

No new data were created.

## Supplementary Materials

The following supporting information can be downloaded at: https://www.mdpi.com/article/10.3390/ijms241914843/s1.

## Abbreviations

Alanine Aminotransferase (ALT), Aspartate transaminase (AST), Bariatric Surgery (BAS), Body Mass Index (BMI), Damage-associated molecular patterns (DAMPs), Dulbecco’s modified Eagle’s medium (DMEM), False Discovery Rates (FDRs), Fetal bovine serum (FBS), Free Fatty Acids (FFA), Gene Expression Omnibus (GEO), Metabolic syndrome (MS), Non-alcoholic fatty liver (NAFL), Non-alcoholic fatty liver disease (NAFLD), Non-alcoholic steatohepatitis (NASH), Overnight (ON), Palmitic acid (PA), Recombinant SPARC (rSPARC), Regular laboratory chow diet (RD), Roswell Park Memorial Institute medium (RPMI), Secreted protein acidic and rich in cysteine (SPARC), Transcripts per million (TPM), Western diet (WD).

## References

[1] Younossi Z.M., Koenig A.B., Abdelatif D., Fazel Y., Henry L., Wymer M. (2016). Global epidemiology of nonalcoholic fatty liver disease-Meta-analytic assessment of prevalence, incidence, and outcomes. Hepatology.

[2] Younossi Z., Tacke F., Arrese M., Sharma B.C., Mostafa I., Bugianesi E., Wong V.W.-S., Yilmaz Y., George J., Fan J. (2019). Global Perspectives on Nonalcoholic Fatty Liver Disease and Nonalcoholic Steatohepatitis. Hepatology.

[3] Hardy T., Oakley F., Anstee Q.M., Day C.P. (2016). Nonalcoholic Fatty Liver Disease: Pathogenesis and Disease Spectrum. Annu. Rev. Pathol. Mech. Dis..

[4] Takahashi Y. (2014). Histopathology of nonalcoholic fatty liver disease/nonalcoholic steatohepatitis. World J. Gastroenterol..

[5] Schuster S., Cabrera D., Arrese M., Feldstein A.E. (2018). Triggering and resolution of inflammation in NASH. Nat. Rev. Gastroenterol. Hepatol..

[6] Mazzolini G., Sowa J.-P., Atorrasagasti C., Kücükoglu Ö., Syn W.-K., Canbay A. (2020). Significance of Simple Steatosis: An Update on the Clinical and Molecular Evidence. Cells.

[7] Loomba R., Friedman S.L., Shulman G.I. (2021). Mechanisms and disease consequences of nonalcoholic fatty liver disease. Cell.

[8] Singh S., Allen A.M., Wang Z., Prokop L.J., Murad M.H., Loomba R. (2014). Fibrosis Progression in Nonalcoholic Fatty Liver vs Nonalcoholic Steatohepatitis: A Systematic Review and Meta-analysis of Paired-Biopsy Studies. Clin. Gastroenterol. Hepatol..

[9] Loomba R., Adams L.A. (2019). The 20% Rule of NASH Progression: The Natural History of Advanced Fibrosis and Cirrhosis Caused by NASH. Hepatology.

[10] Bradshaw A.D., Sage E.H. (2001). SPARC, a matricellular protein that functions in cellular differentiation and tissue response to injury. J. Clin. Investig..

[11] Atorrasagasti C., Onorato A.M., Mazzolini G. (2022). The role of SPARC (secreted protein acidic and rich in cysteine) in the pathogenesis of obesity, type 2 diabetes, and non-alcoholic fatty liver disease. J. Physiol. Biochem..

[12] Ghanemi A., Yoshioka M., St-Amand J. (2020). Secreted Protein Acidic and Rich in Cysteine: Metabolic and Homeostatic Properties beyond the Extracellular Matrix Structure. Appl. Sci..

[13] Kos K., Wilding J.P.H. (2010). SPARC: A key player in the pathologies associated with obesity and diabetes. Nat. Rev. Endocrinol..

[14] Atorrasagasti C., Peixoto E., Aquino J.B., Kippes N., Malvicini M., Alaniz L., Garcia M., Piccioni F., Fiore E.J., Bayo J. (2013). Lack of the Matricellular Protein SPARC (Secreted Protein, Acidic and Rich in Cysteine) Attenuates Liver Fibrogenesis in Mice. PLoS ONE.

[15] Atorrasagasti C., Onorato A., Gimeno M.L., Andreone L., Garcia M., Malvicini M., Fiore E., Bayo J., Perone M.J., Mazzolini G.D. (2019). SPARC is required for the maintenance of glucose homeostasis and insulin secretion in mice. Clin. Sci..

[16] Frizell E., Liu S.-L., Abraham A., Ozaki I., Eghbali M., Sage E.H., Zern M.A. (1995). Expression of SPARC in normal and fibrotic livers. Hepatology.

[17] Onorato A.M., Fiore E., Bayo J., Casali C., Fernandez-Tomé M., Rodríguez M., Domínguez L., Argemi J., Hidalgo F., Favre C. (2021). SPARC inhibition accelerates NAFLD-associated hepatocellular carcinoma development by dysregulating hepatic lipid metabolism. Liver Int..

[18] Mazzolini G., Atorrasagasti C., Onorato A., Peixoto E., Schlattjan M., Sowa J.-P., Sydor S., Gerken G., Canbay A. (2018). SPARC expression is associated with hepatic injury in rodents and humans with non-alcoholic fatty liver disease. Sci. Rep..

[19] Nie J., Bradshaw A.D., Delany A.M., Sage E.H. (2010). Inactivation of SPARC enhances high-fat diet-induced obesity in mice. Connect. Tissue Res..

[20] Ryu S., Sidorov S., Ravussin E., Artyomov M., Iwasaki A., Wang A., Dixit V.D. (2022). The matricellular protein SPARC induces inflammatory interferon-response in macrophages during aging. Immunity.

[21] Gong T., Liu L., Jiang W., Zhou R. (2019). DAMP-sensing receptors in sterile inflammation and inflammatory diseases. Nat. Rev. Immunol..

[22] Kelley N., Jeltema D., Duan Y., He Y. (2019). The NLRP3 inflammasome: An overview of mechanisms of activation and regulation. Int. J. Mol. Sci..

[23] Nolan C.J., Larter C.Z. (2009). Lipotoxicity: Why do saturated fatty acids cause and monounsaturates protect against it?. J. Gastroenterol. Hepatol..

[24] Szabo G., Csak T. (2012). Inflammasomes in liver diseases. J. Hepatol..

[25] Wree A., McGeough M.D., Peña C.A., Schlattjan M., Li H., Inzaugarat M.E., Messer K., Canbay A., Hoffman H.M., Feldstein A.E. (2014). NLRP3 inflammasome activation is required for fibrosis development in NAFLD. J. Mol. Med..

[26] Hoang S.A., Oseini A., Feaver R.E., Cole B.K., Asgharpour A., Vincent R., Siddiqui M., Lawson M.J., Day N.C., Taylor J.M. (2019). Gene Expression Predicts Histological Severity and Reveals Distinct Molecular Profiles of Nonalcoholic Fatty Liver Disease. Sci. Rep..

[27] Bendtsen J.D., Jensen L.J., Blom N., von Heijne G., Brunak S. (2004). Feature-based prediction of non-classical and leaderless protein secretion. Protein Eng. Des. Sel..

[28] Enooku K., Tsutsumi T., Kondo M., Fujiwara N., Sasako T., Shibahara J., Kado A., Okushin K., Fujinaga H., Nakagomi R. (2019). Hepatic FATP5 expression is associated with histological progression and loss of hepatic fat in NAFLD patients. J. Gastroenterol..

[29] Park A., Choi S.J., Park S., Kim S.M., Lee H.E., Joo M., Kim K.K., Kim D., Chung D.H., Im J.B. (2022). Plasma Aldo-Keto Reductase Family 1 Member B10 as a Biomarker Performs Well in the Diagnosis of Nonalcoholic Steatohepatitis and Fibrosis. Int. J. Mol. Sci..

[30] Shi L., Guo S., Zhang S., Gao X., Liu A., Wang Q., Zhang T., Zhang Y., Wen A. (2020). Glycyrrhetinic acid attenuates disturbed vitamin a metabolism in non-alcoholic fatty liver disease through AKR1B10. Eur. J. Pharmacol..

[31] Yang F., Ni B., Lian Q., Qiu X., He Y., Zhang Q., Zou X., He F., Chen W. (2023). Key genes associated with non-alcoholic fatty liver disease and hepatocellular carcinoma with metabolic risk factors. Front. Genet..

[32] Brekken R.A., Sage E. (2001). SPARC, a matricellular protein: At the crossroads of cell-matrix communication. Matrix Biol..

[33] Kato Y., Lewalle J.-M., Baba Y., Tsukuda M., Sakai N., Baba M., Kobayashi K., Koshika S., Nagashima Y., Frankenne F. (2001). Induction of SPARC by VEGF in Human Vascular Endothelial Cells. Biochem. Biophys. Res. Commun..

[34] Peixoto E., Atorrasagasti C., Aquino J.B., Militello R., Bayo J., Fiore E., Piccioni F., Salvatierra E., Alaniz L., García M.G. (2014). SPARC (secreted protein acidic and rich in cysteine) knockdown protects mice from acute liver injury by reducing vascular endothelial cell damage. Gene Ther..

[35] Atorrasagasti C., Aquino J.B., Hofman L., Alaniz L., Malvicini M., Garcia M., Benedetti L., Friedman S.L., Podhajcer O., Mazzolini G. (2011). SPARC downregulation attenuates the profibrogenic response of hepatic stellate cells induced by TGF-β_1_and PDGF. Am. J. Physiol. Liver Physiol..

[36] Nakatani K., Seki S., Kawada N., Kitada T., Yamada T., Sakaguchi H., Kadoya H., Ikeda K., Kaneda K. (2002). Expression of SPARC by activated hepatic stellate cells and its correlation with the stages of fibrogenesis in human chronic hepatitis. Virchows Arch..

[37] Chaly Y., Fu Y., Marinov A., Hostager B., Yan W., Campfield B., Kellum J.A., Bushnell D., Wang Y., Vockley J. (2014). Follistatin-like protein 1 enhances NLRP3 inflammasome-mediated IL-1β secretion from monocytes and macrophages. Eur. J. Immunol..

[38] Cai C., Zhu X., Li P., Li J., Gong J., Shen W., He K. (2017). NLRP3 Deletion Inhibits the Non-alcoholic Steatohepatitis Development and Inflammation in Kupffer Cells Induced by Palmitic Acid. Inflammation.

[39] Csak T., Ganz M., Pespisa J., Kodys K., Dolganiuc A., Szabo G. (2011). Fatty acid and endotoxin activate inflammasomes in mouse hepatocytes that release danger signals to stimulate immune cells. Hepatology.

[40] de Haan J.J., Smeets M.B., Pasterkamp G., Arslan F. (2013). Danger signals in the initiation of the inflammatory response after myocardial infarction. Mediat. Inflamm..

[41] Frevert C.W., Felgenhauer J., Wygrecka M., Nastase M.V., Schaefer L. (2018). Danger-Associated Molecular Patterns Derived From the Extracellular Matrix Provide Temporal Control of Innate Immunity. J. Histochem. Cytochem..

[42] Ribeiro M.d.C., Szabo G. (2022). Role of the Inflammasome in Liver Disease. Annu. Rev. Pathol. Mech. Dis..

[43] Nie J., Sage E.H. (2009). SPARC functions as an inhibitor of adipogenesis. J. Cell Commun. Signal..

[44] Blazejewski S., Le Bail B., Boussarie L., Blanc J.F., Malaval L., Okubo K., Saric J., Bioulac-Sage P., Rosenbaum J. (1997). Osteonectin (SPARC) expression in human liver and in cultured human liver myofibroblasts. . Am. J. Pathol..

[45] Larsen F.T., Hansen D., Terkelsen M.K., Bendixen S.M., Avolio F., Wernberg C.W., Lauridsen M.E., Grønkjaer L.L., Jacobsen B.G., Klinggaard E.G. (2022). Stellate cell expression of SPARC-related modular calcium-binding protein 2 is associated with human non-alcoholic fatty liver disease severity. JHEP Rep..

[46] Inzaugarat M.E., Johnson C.D., Holtmann T.M., McGeough M.D., Trautwein C., Papouchado B.G., Schwabe R., Hoffman H.M., Wree A., Feldstein A.E. (2019). NLR Family Pyrin Domain-Containing 3 Inflammasome Activation in Hepatic Stellate Cells Induces Liver Fibrosis in Mice. Hepatology.

[47] Mridha A.R., Wree A., Robertson A.A., Yeh M.M., Johnson C.D., Van Rooyen D.M., Haczeyni F., Teoh N.C.-H., Savard C., Ioannou G.N. (2017). NLRP3 inflammasome blockade reduces liver inflammation and fibrosis in experimental NASH in mice. J. Hepatol..

[48] Park I., Kim N., Lee S., Park K., Son M.-Y., Cho H.-S., Kim D.-S. (2023). Characterization of signature trends across the spectrum of non-alcoholic fatty liver disease using deep learning method. Life Sci..

[49] Rosso N., Stephenson A.M., Giraudi P.J., Tiribelli C. (2021). Diagnostic management of nonalcoholic fatty liver disease: A transformational period in the development of diagnostic and predictive tools—A narrative review. Ann. Transl. Med..

[50] Sumida Y., Nakajima A., Itoh Y. (2014). Limitations of liver biopsy and non-invasive diagnostic tests for the diagnosis of nonalcoholic fatty liver disease/nonalcoholic steatohepatitis. World J. Gastroenterol..

[51] Brunt E.M. (2017). Nonalcoholic fatty liver disease and the ongoing role of liver biopsy evaluation. Hepatol. Commun..

[52] Lemmer P., Selbach N., Baars T., Porsch-Özcürümez M., Heider D., Canbay A., Sowa J.-P. (2021). Transaminase Concentrations Cannot Separate Non-Alcoholic Fatty Liver and Non-Alcoholic Steatohepatitis in Morbidly Obese Patients Irrespective of Histological Algorithm. Dig. Dis..

[53] Schindhelm R.K., Diamant M., Dekker J.M., Tushuizen M.E., Teerlink T., Heine R.J. (2006). Alanine aminotransferase as a marker of non-alcoholic fatty liver disease in relation to type 2 diabetes mellitus and cardiovascular disease. Diabetes/Metab. Res. Rev..

[54] Fiore E.J., Bayo J.M., Garcia M.G., Malvicini M., Lloyd R., Piccioni F., Rizzo M., Peixoto E., Sola M.B., Atorrasagasti C. (2015). Mesenchymal stromal cells engineered to produce IGF-I by recombinant adenovirus ameliorate liver fibrosis in mice. Stem Cells Dev..

[55] Win S., Than T.A., Le B.H.A., García-Ruiz C., Fernandez-Checa J.C., Kaplowitz N. (2015). Sab (Sh3bp5) dependence of JNK mediated inhibition of mitochondrial respiration in palmitic acid induced hepatocyte lipotoxicity. J. Hepatol..

[56] Ibrahim S.H., Hirsova P., Malhi H., Gores G.J. (2015). Animal Models of Nonalcoholic Steatohepatitis: Eat, Delete, and Inflame. Dig. Dis. Sci..

[57] Lieber C.S., Leo M.A., Mak K.M., Xu Y., Cao Q., Ren C., Ponomarenko A., DeCarli L.M. (2004). Model of nonalcoholic steatohepatitis. Am. J. Clin. Nutr..

[58] Kleiner D.E., Brunt E.M., Van Natta M., Behling C., Contos M.J., Cummings O.W., Ferrell L.D., Liu Y.-C., Torbenson M.S., Unalp-Arida A. (2005). Design and validation of a histological scoring system for nonalcoholic fatty liver disease. Hepatology.

[59] Sumida Y., Yoneda M., Hyogo H., Itoh Y., Ono M., Fujii H., Eguchi Y., Suzuki Y., Aoki N., Kanemasa K. (2012). Validation of the FIB4 index in a Japanese nonalcoholic fatty liver disease population. BMC Gastroenterol..

