# Supplementary material for: Hepatic SPARC Expression Is Associated with Inflammasome Activation during the Progression of Non-Alcoholic Fatty Liver Disease in Both Mice and Morbidly Obese Patients
Source: Int J Mol Sci. 2023 Oct 2;24(19):14843. doi: 10.3390/ijms241914843 (PMC10573696; doi:10.3390/ijms241914843)
Supplement: Supplementary file 1 [file ijms-24-14843-s001.zip › ijms-2617295 Supplementary Table S1.pdf]

Supplementary Table S1. Demographic and histopathological characteristics of the public database GSE130970.

	Variable		Healthy	Fatty liver	NASH borderline	NASH
GSE130970	n (Total = 78)		4 (5.1%)	14 (17.9%)	34 (43.6%)	26 (33.3%)
	Sex	M	1 (25%)	7 (50%)	13 (38%)	9 (35%)
		F	3 (75%)	7 (50%)	21 (62%)	17 (65%)
	Age (mean) (SD)	M	26 (0.0)	46.6 (14.9)	44.9 (10.0)	47.1 (14.7)
		F	62 (2.0)	54.3 (9.6)	49.5 (11.5)	57.5 (9.5)
	**NAFLD activity score**		**0**	**1 & 2**	**3 & 4**	**5, 6 & 7**
	Steatosis grade	0	4 (100%)	4 (29%)	0	0
		1	0	9 (64%)	18 (53%)	2 (8%)
		2	0	1 (7%)	13 (38%)	13 (50%)
		3	0	0	3 (9%)	11 (42%)
	Lobular inflammation grade	0	4 (100%)	4 (29%)	1 (3%)	0
		1	0	9 (64%)	30 (88%)	18 (69%)
		2	0	1 (7%)	3 (9%)	8 (31%)
	Cytological ballooning grade	0	4 (100%)	13 (93%)	12 (35%)	1 (4%)
		1	0	1 (7%)	15 (44%)	7 (27%)
		2	0	0	7 (21%)	18 (69%)
	**Fibrosis stage**					
		0	4 (100%)	10 (71.5%)	11 (32%)	1 (4%)
		1	0	3 (21.5%)	13 (38%)	12 (46%)
		2	0	1 (7%)	4 (12%)	4 (15%)
		3	0	0	5 (15%)	8 (31%)
		4	0	0	1 (3%)	1 (4%)

Demographic data of patients with NAFLD from RNAseq database GSE130970 (http://www.ncbi.nlm.nih.gov/geo/). The table shows number of patients per group (n), sex (M: male, F: female), and age. Histopathological variables provide NAFLD activity score (steatosis grade, lobular inflammation grade and cytological ballooning grade), and fibrosis stage. Results given as mean ± SD or n (%).
