# Supplementary material for: Hepatic SPARC Expression Is Associated with Inflammasome Activation during the Progression of Non-Alcoholic Fatty Liver Disease in Both Mice and Morbidly Obese Patients
Source: Int J Mol Sci. 2023 Oct 2;24(19):14843. doi: 10.3390/ijms241914843 (PMC10573696; doi:10.3390/ijms241914843)
Supplement: Supplementary file 1 [file ijms-24-14843-s001.zip › ijms-2617295 Supplementary Table S2.pdf]

Supplementary Table S2. Demographic, histopathological and biochemical comparisons between low and high subgroup patients.

	SPARC		CASP1		IL1-b		AKR1B10		FABP5	
	Low	High	Low	High	Low	High	Low	High	Low	High
**n**	36	29	33	32	36	28	35	30	32	33
**Sex**	M:21 F:15	M:14 F:15	M:16 F:16	M:13 F:19	M:26 F:16	M:14 F:14	M:24 F:11	M:11 F:19	M:18 F:14	M:17 F:16
**Age (SD)**	46.4 (14.4)	46.9 (11.3)	47 (10)	45.9 (9.5)	46.75 (8.7)	46.2 (11.1)	47.7 (9.3)	45 (10.2)	46.25 (9.3)	46.67 (10.3)
**IMC (SD)**	40.8 (5.8)	41.97 (8.6)	40.61 (5.5)	42.1 (9.5)	39.81 (5.3)	43.5 (8.6)	41.3 (6.8)	41.3 (7.6)	40.87 (5.6)	41.76 (8.4)
**NAFLD score**	3 (7)	3.5 (7)	3.5 (7)	3 (7)	4 (7)	3 (7)	4 (7)	3 (6)	4 (6)	3 (7)
**Fibrosis score**	1 (4)	1 (3)	1 (4)	1 (4)	1 (4)	1 (3)	1 (4)	1 (3)	1 (4)	1 (3)
**AlbG (g/dL)**	3.9±0.1	4.0±0.1	4.0±0.1	3.9±0.1	4.0±0.1	3.9±0.1	4.1±0.1	3.8±0.1	4.1±0.1	3.9±0.1
**AlkP (U/L)**	46.7±4.3	55.5±4.7	53.9±4.2	53.6±4.8	53.9±4.8	52.9±4.3	52.4±4.4	55.5±4.6	52.3±3.8	55.1±5.1
**ALT (U/L)**	12.2±3.7	12.8±1.5	20.7±3.6	12.4±1.5	19.6±3.4	13.9±2.2	19.0±2.4	13.6±3.5	22.0±3.6	11.3±1.5
**AST (U/L)**	32.7±5	44.8±7.5	46.5±6.6	39.0±6.0	52.9±7.6	32.7±3.5	47.7±5.6	36.4±7.2	50.0±6.5	35.7±5.9
**BilD (mg/dL)**	0.2±0.1	0.2±0.0	0.3±0.0	0.2±0.0	0.2±0.0	0.3±0.0	0.2±0.0	0.3±0.0	0.2±0.0	0.3±0.0
**BiliT (mg/dL)**	0.5±0.1	0.6±0.0	0.6±0.1	0.6±0.1	0.6±0.1	0.6±0.1	0.6±0.1	0.6±0.1	0.6±0.1	0.6±0.1
**Chol (mg/dL)**	140.1±6.6	140.7±6.1	146.6±5.9	142.3±6.9	148.5±6.5	141.2±6.3	147.3±5.3	140.9±7.8	152.4±6.0	136.7±6.5
**GluC (mg/dL)**	111.4±6.9	111.9±4.8	118.6±6.1	113.7±5.8	116.9±5.9	116.2±6.3	120.9±5.7	110.1±6.1	126.8±6.2	105.7±5.1
**TP (g/dL)**	7.2±0.2	7.6±0.3	7.3±0.3	7.4±0.3	7.6±0.3	7.1±0.1	7.7±0.2	7.0±0.3	7.5±0.2	7.2±0.3
