# Supplementary material for: Hepatic SPARC Expression Is Associated with Inflammasome Activation during the Progression of Non-Alcoholic Fatty Liver Disease in Both Mice and Morbidly Obese Patients
Source: Int J Mol Sci. 2023 Oct 2;24(19):14843. doi: 10.3390/ijms241914843 (PMC10573696; doi:10.3390/ijms241914843)
Supplement: Supplementary file 1 [file ijms-24-14843-s001.zip › ijms-2617295 Supplementary Table S3.pdf]

Supplementary Table S3. Primers sequences information.

Primers	Sequence 5´ → 3´
mGapdh – Fw	(DNA)- GGG GCT GCC CAG AAC ATC AT
mGapdh – Rv	(DNA)- GCC TGC TTC ACC ACC TTC TTG
mSparc – Fw	(DNA)- CCA CAC GTT TCT TTG AGA CC
mSparc – Rv	(DNA)- GAT GTC CTG CTC CTT GAT GC
mNlrp3 – Fw	(DNA)- TGC TCT TCA CTG CTA TCA AGC CCT
mNlrp3 – Rv	(DNA)- ACA AGC CTT TGC TCC AGA CCC TAT
mCaspase1 – Fw	(DNA)- TGG CTT CTT ATT GGC ACG AT
mCaspase1 – Rv	(DNA)- TGG TCT TGT GAC TTG GAG GA
mIl-1β – Fw	(DNA)- TGA CAG TGA TGA GAA TGA CCT GTT C
mIl-1β – Rv	(DNA)- TTG GAA GCA GCC CTT CAT CT
18s – Fw	(DNA)- ACC GCA GCT AGG AAT AAT GGA
18s – Rv	(DNA)- GCC TCA GTT CCG AAA ACC A
hSPARC – Fw	(DNA)- AAA CCG AAG AGG AGG TGG TG
hSPARC – Rv	(DNA)- GCA AAG AAG TGG CAG GAA GA
hNLRP3 – Fw	(DNA)- GAT CTT CGC TGC GAT CAA CAG
hNLRP3 – Rv	(DNA)- TCA ATG CTG TCT TCC TGG CA
hCASPASE1 – Fw	(DNA)- GTG CAG GAC AAC CCA GCT AT
hCASPASE1 – Rv	(DNA)- TGC GGC TTG ACT TGT CCA TT
hIL-1β – Fw	(DNA)-CCT CCA GGG ACA GGA TAT GGA
hIL-1β – Rv	(DNA)-TTT CAA CAC GCA GGA CAG GTA
hAKR1B10 – Fw	(DNA)- GTA ACG TGT TGC AAT CCT CT
hAKR1B10 – Rv	(DNA)- GAC ATG AGT GGA GGT AGT CA
hFABP5 – Fw	(DNA)- TTT GAT GAA TAC ATG AAG GAG CTA
hFABP5 – Rv	(DNA)- GTG ATG ATA CAA TCT GGC TTG G
